# Impact of COVID-19 on Surgical Procedural Utilization

**DOI:** 10.7759/cureus.82772

**Published:** 2025-04-22

**Authors:** Timothy E Nehila, Bilal Koussayer, Salvatore Docimo, Christopher G DuCoin

**Affiliations:** 1 Surgery, University of South Florida Health Morsani College of Medicine, Tampa, USA

**Keywords:** covid19, elective, non-elective, pandemic, surgery, utilization

## Abstract

Background

This study examines the impact of COVID-19 infection waves on the healthcare utilization of elective procedures versus non-elective procedures.

Methods

Eligible encounters were classified into simple/elective (elective) and cancer/complex (non-elective) groups based on the ICD-10-CM diagnosis codes. Procedure-specific volumes were used to evaluate healthcare utilization.

Results

Compared with the non-elective cohort, the elective cohort showed a greater dip (93% and 58% of the baseline volumes in March and April 2020 vs. 70% and 18% respectively, p-value = 0.0001). Similar patterns were identified for each successive wave of the pandemic.

Conclusions

During each of the first four waves of the pandemic, elective procedure volumes both fell and recovered at higher relative rates when compared with non-elective procedure volumes. Throughout the pandemic, there was a trend toward less cancelation of both elective and non-elective procedures with each successive wave. Our findings characterize the pandemic as a cyclic disease that surgical specialties are learning to cope with over time.

## Introduction

The COVID-19 pandemic was officially declared by the World Health Organization in March 2020 [1]. What followed was a shutdown of virtually all non-essential organizations in an attempt to contain the pandemic. The healthcare system was not without impact, as we observed the discharge of a majority of inpatients and the cancelation of surgical cases to make room for COVID-19 patients requiring acute and intensive care [2]. With an increase in demand for essential medical care and a decrease in the supply of medical resources due to global supply chain unrest, the world experienced a healthcare resource crisis [2]. Therefore, it was not surprising that there was a 50% decrease in elective procedure volume during the first month of the pandemic [3].

Elective procedures are non-emergent procedures undertaken by patients to improve their health or appearance rather than to treat an acute illness. Unfortunately, postponing these procedures delays treatment that can have a profound impact on quality of life and the amelioration of future chronic illness. This is seen in fields such as plastic surgery, for reconstruction of certain acquired deformities due to cancer lesions, orthopedics, for joint replacements that help patients avoid sedentary lifestyles, and bariatrics, for gastric bypass surgeries that help patients lose weight and decrease the risk for diseases such as diabetes and coronary artery disease [4].

In the current literature, the impact of COVID-19 on the safety and management of elective procedures such as gastric bypass, hysterectomy, non-cancer colorectal surgery, and simple hernia repair has been well described and exhausted [5-8]. Yet the literature lacks data about fluctuating elective and non-elective procedure volume in the United States during the beginning of the pandemic and throughout subsequent outbreaks. Although healthcare providers experienced an initial shutdown in March 2020, the impact of supply shortages continued through the end of the year [9]. In the United States, multiple subsequent waves of COVID-19 heaved after the initial outbreak. From July to August 2020, the summer surge was attributed to the relaxation of social distancing measures and the reopening of business and public spaces that had been closed during the early months of the pandemic [10]. In December 2020 and January 2021, the winter wave saw the spread of the virus due to the increased number of social gatherings and frequency of travel during the holiday months [11]. During the summer of 2021, the Delta wave also caused a resurgence of COVID-19 cases due to mutations in the spike protein yielding a more transmissible and virulent Delta variant [12]. Lastly, in December of 2021, the Omicron wave was also due to a spike protein mutation, and although less virulent, it was correlated with higher hospitalization rate in children [13]. Of note, vaccines became widely available in the early months of 2021 with over 50% of Americans being vaccinated by the summer of 2021 before the Delta and Omicron waves [14]. Because of the dynamic nature of the COVID-19 pandemic, it is important to describe and analyze its impact on elective and non-elective procedures not as a single event, but as a cyclical battle that has been evolving since the pandemic’s inception. The objective of this study is to measure and compare fluctuations in the volume of selected elective and non-elective surgical procedures across each of the first four waves of the COVID-19 pandemic in the United States.

## Materials and methods

Data source

PINC AITM Healthcare Database (PHD) contains patient-level administrative claims data from more than 260 million unique patients from approximately 800 US hospitals. It roughly represents 20% of annual US inpatient and outpatient encounters. It includes patient demographics, hospital characteristics, payer information, International Classification of Diseases, Tenth Revision (ICD-10) primary and secondary diagnosis and procedure codes, and admission and discharge months for each encounter. Hospitals and healthcare system records within PHD are deemed as national representative and Health Insurance Portability and Accountability Act (HIPAA)-compliant pursuant to 45 CFR 164.514(b)(1) through the “Expert Determination” method. Therefore, institutional review board or ethics committee approval was not required for this study [15].

Study cohort

We focused on six procedures including bariatric, colorectal, lung lobectomy, hysterectomy, incisional/ventral hernia, and inguinal hernia repairs. Patients were identified by ICD-10 procedure codes and/or Current Procedural Terminology (CPT) codes (Table 2, Appendix). The PHD database was reviewed from January 1, 2019 through December 31, 2021 to identify patients who underwent the six procedures. Specifically for bariatric populations, the qualifying ICD-10-CM diagnosis code, E66.01 (Morbid (severe) obesity due to excess calories), must be present in the hospital encounter in addition to the bariatric procedure codes to be included in the study. Once the eligible encounters were identified, they were then classified into simple/elective (elective) and cancer/complex (non-elective) groups based on the ICD-10-CM diagnosis codes listed in Table 3 in the Appendix.

Consistent contributing provider

As the study objective is to evaluate the impact, it is meaningful to compare the same providers before and after the COVID-19 pandemic onset. Therefore, we only included encounters from hospitals that constantly contributed data across the study period. More specifically, a consistent contributing provider is defined as a hospital that had encounters with procedures of interest in all three years (2019 - 2021) within a given month.

Outcomes

Procedure-specific volumes were used to evaluate healthcare utilization, which were calculated in each month across the study time span (2019 - 2021). We used the 2019 volumes as a pre-COVID-19 baseline and calculated the proportion of 2020 and 2021 volumes to the baseline as the recovery rate (equation below). One advantage of adapting the recovery rate is that the procedure volume seasonal fluctuations are accounted for.

Recovery Rate (month) = [Follow-up Volume(month)/Baseline Volume(month)] x 100 (%)

Statistical analysis

Categorical variables were reported as frequencies and percentages with a chi-square test or Fisher's exact test to examine the statistical significance. Since the study period was 2019 - 2021 and used 2019 as a baseline, recovery rates were calculated from only 24 months (years of 2020 and 2021). To account for the small sample size, the impact of the COVID-19 pandemic on the surgical volume recovery rate between elective and non-elective procedure cohorts was examined using an interrupted time series analysis model incorporating a Type II Sum Square ANCOVA Lagged Dependent Variable and variance-centric approach [16].

The data extraction, recovery rate calculation, and interrupted time series analysis were executed using SAS software, version 9.4 (SAS Institute, Cary, NC) and R studio 2022.12.0 [17] and trend analysis was plotted using Microsoft Excel (Microsoft Corporation, Redmond, USA) where the recovery rates were plotted along a time axis to demonstrate recovery trends over time.

## Results

**Figure 1:**
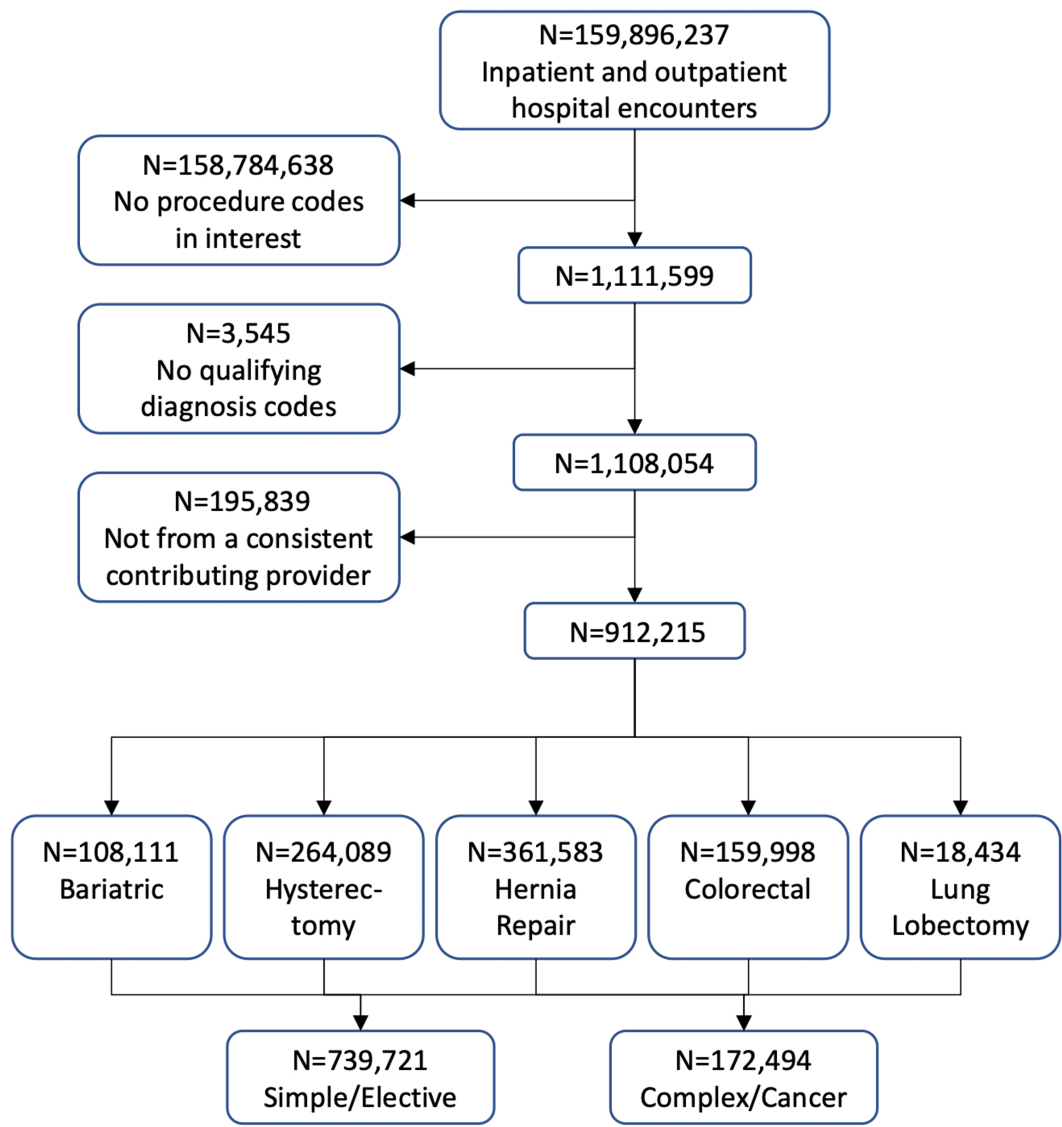
Population selection flow from PHD 2019 to 2021. PHD: PINC AITM Healthcare Database

Of 160 million inpatient and outpatient encounters, 1.1 million were identified to have the procedures of interest in the 2019 - 2021 PHD database. After excluding those without qualifying ICD-10-CM diagnosis codes (3,545 encounters) and those that were not from consistent contributing providers (195,839 encounters), 912,215 encounters comprised bariatric (11.8%), hysterectomy (29.0%), hernia repair (40.0%), colorectal (17.5%), and lung lobectomy (2%) procedures (Figure 1). Furthermore, 81.1% of all the eligible encounters were classified as simple/elective cases and 18.9% were complex or cancer (non-elective) cases.

In general, the complex or cancer populations were older than the simple/elective populations, where approximately 45% of the complex or cancer cases were over the age of 65 compared to 77% of the simple/elective cases who were younger than 65 (p-value < 0.0001). With such a difference in age, a higher percentage of Medicare insured populations were expected in the complex or cancer populations (48.6% versus 27.1%, p-value < 0.0001) while the majority (51.2%) of the simple/elective populations were under commercial insurances (Table 1).

**Table 1:** Patient demographic and characteristic summary by surgical type in count (percent) format.

	Overall	Simple/Elective	Cancer/ Complex	Chi-square
	(n = 912,215)	(n = 739,721)	(n = 172,494)	p-value
Age Group				
< 20	17,289 (1.9%)	16,362 (2.2%)	927 (0.5%)	< .0001
20 - 65	647,681 (71.0%)	553,878 (74.9%)	93,803 (54.4%)	
> 65	247,245 (27.1%)	169,481 (22.9%)	77,764 (45.1%)	
Gender				
Female	543,167 (59.5%)	446,961 (60.4%)	96,206 (55.8%)	< .0001
Male	369,048 (40.5%)	292,760 (39.6%)	76,288 (44.2%)	
Payer Mix				
Medicare	283,925 (31.1%)	200,148 (27.1%)	83,777 (48.6%)	< .0001
Medicaid	127,354 (14.0%)	109,545 (14.8%)	17,809 (10.3%)	
Commercial	438,809 (48.1%)	378,589 (51.2%)	60,220 (34.9%)	
Other	62,127 (6.8%)	51,439 (7.0%)	10,688 (6.2%)	
Surgery Approach				
MIS	602,135 (66.0%)	508,693 (68.8%)	93,442 (54.2%)	< .0001
Open	310,080 (34.0%)	231,028 (31.2%)	79,052 (45.8%)	
Urban Hospital	791,378 (86.8%)	639,459 (86.4%)	151,919 (88.1%)	< .0001
Teaching Hospital	430,881 (47.2%)	341,879 (46.2%)	89,002 (51.6%)	< .0001
Hospital Bed Size				
< 100	63,685 (7.0%)	54,467 (7.4%)	9,218 (5.3%)	< .0001
100 - 299	291,233 (31.9%)	242,454 (32.8%)	48,779 (28.3%)	
300+	557,297 (61.1%)	442,800 (59.9%)	114,497 (66.4%)	
Provider Division				
East North Central	154,901 (17.0%)	125,948 (17.0%)	28,953 (16.8%)	< .0001
East South Central	81,849 (9.0%)	66,990 (9.1%)	14,859 (8.6%)	
Middle Atlantic	103,626 (11.4%)	83,912 (11.3%)	19,714 (11.4%)	
Mountain	78,790 (8.6%)	64,982 (8.8%)	13,808 (8.0%)	
New England	26,751 (2.9%)	21,652 (2.9%)	5,099 (3.0%)	
Pacific	80,015 (8.8%)	63,042 (8.5%)	16,973 (9.8%)	
South Atlantic	242,417 (26.6%)	195,539 (26.4%)	46,878 (27.2%)	
West North Central	53,675 (5.9%)	44,156 (6.0%)	9,519 (5.5%)	
West South Central	90,191 (9.9%)	73,500 (9.9%)	16,691 (9.7%)	

Both simple/elective and complex or cancer procedures in the study population were conducted in similar healthcare facilities where 86% were in urban areas, 47% provided medical education and 60% had 300 or more bed capacity. Geographically, the procedures took place in all nine US divisions with slightly higher numbers in East North Central (17%) and South Atlantic (27%) divisions (Table 1).

**Figure 2:**
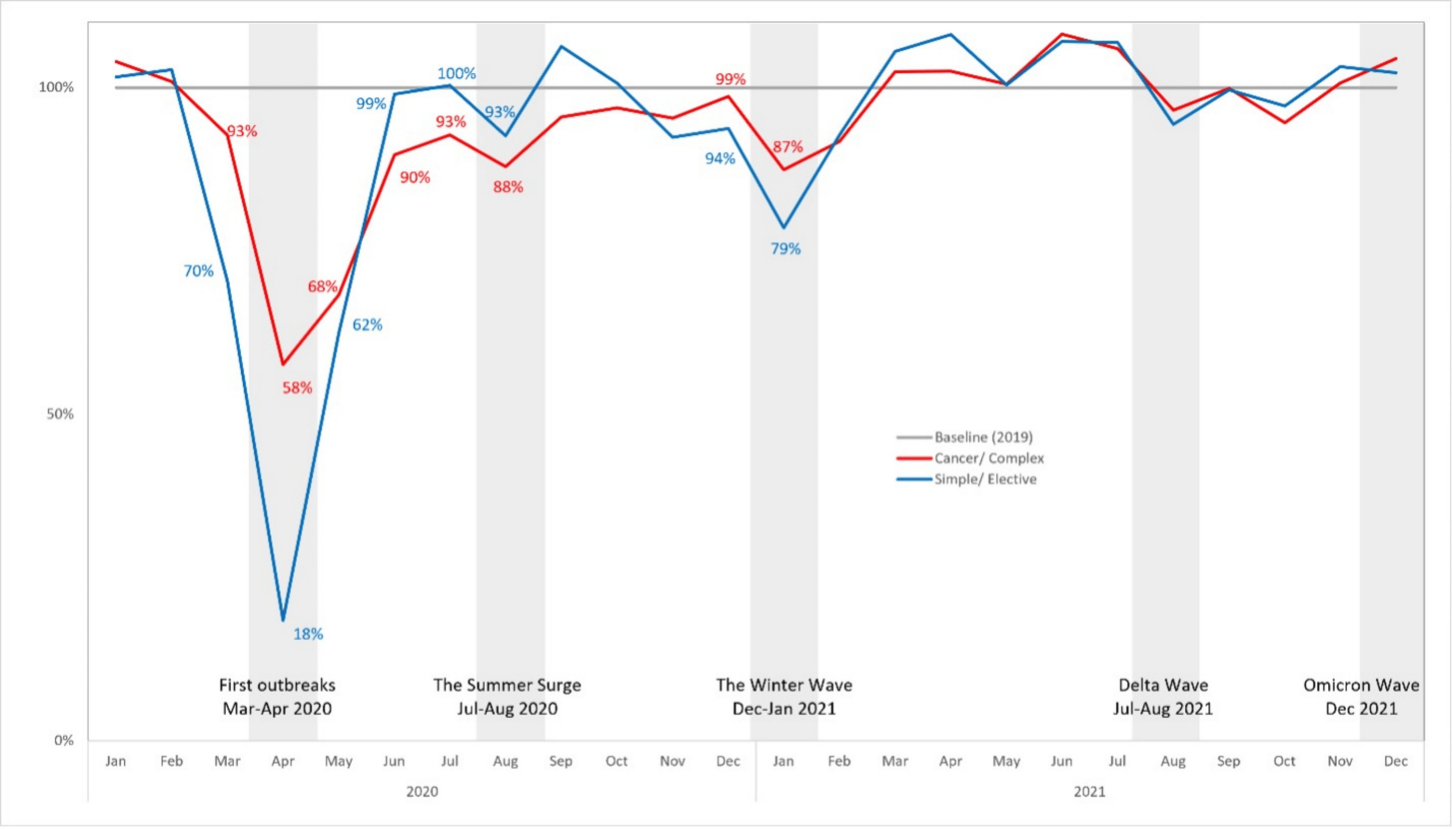
Procedure volume recovery rate by procedure types (baseline: 2019 volumes).

As depicted in Figure 2, both simple/elective and complex or cancer (non-elective) procedure volumes showed a significant dip when the pandemic started in March 2020 and hit the bottom in April 2020 (First Outbreak). The interrupted time series analysis showed that compared with the complex/cancer cohort, the simple/elective cohort showed a greater dip (93% and 58% of the baseline volumes in March and April 2020 vs. 70% and 18% respectively, p-value = 0.0001). Thereafter, the simple/elective procedure volumes recovered to baseline levels in June 2020 (99%). The complex or cancer procedure volumes showed a slower recovery, as they were at 90% in June 2020.

The pandemic reached another peak in July-August 2020 (Summer Surge). Like the First Outbreak, the simple/elective procedure suffered a greater impact (recovery rate dropped 7% in August 2020 from 100% in July) than the complex or cancer procedure (dropped 5% in the same period from 93%) in the Summer Surge. A similar pattern was observed again in December 2020-January 2021 when the Winter Wave hit. In that period, the simple/elective procedure recovery rate decreased 15% in January 2021 from 94% in December 2020, while the complex or cancer procedure decreased 12% from 99% in December 2020.

Since then, the simple/elective and complex or cancer procedures recovered and exceeded the 2019 baseline until July-August 2021, when the Delta variant began. Both the procedures dropped below the baseline, but once again, the simple/elective procedures showed a higher decrease than the complex or cancer procedure from July to August 2021 (13% and 9%, respectively). Afterward, both simple/elective and complex or cancer procedure volumes reached the pre-COVID-19 level in December 2021, and the Omicron wave did not seem to have such an impact as previous waves.

## Discussion

The aim of this study was to describe the dynamic impact of the COVID-19 pandemic on relative rates of elective and non-elective surgical procedures. We hypothesized that elective procedure volumes would decrease faster and to a greater extent than non-elective procedure volumes. In this paper, we show that during each of the first four waves of the pandemic, simple/elective procedure volumes both fell and recovered at higher relative rates when compared with complex or cancer (non-elective) procedure volumes. Additionally, we demonstrate a trend towards attenuated decline of both elective and non-elective procedure volumes with each phase of the pandemic, culminating in the most recent Omicron wave, where we saw no impact on either elective or non-elective procedure volume. Interestingly, our data also show that the Medicaid population had higher recovery rates in elective and non-elective procedures than other payers. Overall, our findings characterize the COVID-19 pandemic as a cyclic disease that surgical specialties are learning to cope with over time.

First, our findings validate the hypothesis that elective procedure volumes would fall at faster rates and to lower relative values than non-elective procedures. This result is unsurprising given the widespread national and state-level recommendations and/or explicit guidelines for elective surgical procedures during the pandemic. The first of such recommendations came down from the Centers for Medicare and Medicaid Services (CMS) on March 18, 2020, and suggested that all elective surgeries, non-essential medical, surgical, and dental procedures be delayed. Along with this recommendation, the CMS provided hospitals and clinicians with specific examples to guide whether to postpone a given surgery [18]. Although organizations like CMS dispensed early national recommendations, in most states, the ultimate decision whether to cancel elective procedures was left up to individual hospital systems and surgeons. The one exception to this trend was in Maryland, where they ordered the restriction of elective medical procedures enforced by punishments including up to one year imprisonment, a fine up to $5000, or both [18]. Although there was never an explicit or recommended ban on non-elective procedures during the pandemic, our data also show declines in the procedure volume for these surgeries. This relative regression was likely a symptom of strict stay-at-home orders and the reluctance of patients to visit the hospital for fear of contracting COVID-19 infection. Correspondingly, one study found that the number of average daily ED visits decreased by 20% during the pandemic [19].

The overall trend in both elective and non-elective surgery volume was towards less regression with each wave of the pandemic. Before discussing some of the reasons for this trend, it is important to define the two principal motives behind elective procedure cancelation: concerns over hospital bandwidth and concern for patient exposure to COVID-19. Early in the pandemic, when faced with an unprecedented novel virus, it made sense to exercise caution and conserve as many resources as possible for COVID-19 patients. However, as the pandemic progressed, physicians and researchers turned their attention towards understanding the virus and began to uncover ways to address the concerns that led to procedure cancelation. One study that tackled the question of hospital bandwidth concluded that elective outpatient surgery consumed negligible hospital resources and should not be considered a threat in the setting of high demand by critically ill COVID-19 patients [20]. Concerns for patient exposure were addressed by both surgeons and the healthcare system at large. First, incredible strides were made by the US healthcare system to develop and administer vaccinations against COVID-19 [14]. Concurrently, research was conducted to investigate steps that prevent transmission in the operating room, leading to safer practices that allowed more elective and non-elective cases to be scheduled [21]. These studies highlight a data-driven response to the COVID-19 pandemic by the US healthcare system and surgical specialties. Taken together with ambiguous recommendations for elective surgery from national and state organizations, it is unsurprising that procedure volumes were less affected with each successive wave of the pandemic.

Lastly, though not presented in this paper, our data suggested that the Medicaid population had higher recovery rates of elective and non-elective procedures than other payers. It will be interesting to further investigate this trend in relation to Medicaid expansion in the Affordable Care Act and the temporary continuous enrollment provision created by the Families First Coronavirus Response Act (FFCRA) [22]. It will also be interesting to investigate the long-term impact of increased access to surgical procedures by Medicaid payers, including rates of chronic disease in this population.

Limitations

As is the nature of administrative claims data, the PHD database is subject to clinical coding errors introduced during provider submissions to PINC AI™, although the proportion is expected to be small. In addition, the PHD database only covers facility claims from inpatient and outpatient hospital settings, excluding other sources such as independent ambulatory surgical centers (ASCs) or office-based procedures. This limits the comprehensiveness of procedure volume estimates and may underrepresent certain specialties or regions. Furthermore, there may be potential biases or unmeasured confounding factors, such as regional differences in COVID-19 policy, hospital resource availability, or timing of elective surgery restrictions, that could influence procedure volumes independently of infection waves. These factors were not fully controlled for in the present analysis and may have affected observed trends. The generalizability of our findings may also be limited if the dataset lacks sufficient geographic or demographic diversity or if the sample size for certain procedures is small. Additionally, the reliance on administrative data introduces constraints related to the granularity of clinical detail and patient-level characteristics, which could influence interpretation. Lastly, although we are transparent about the methods used, we acknowledge that full replicability is limited by the absence of publicly available code lists and specific ICD-10 definitions.

## Conclusions

During each of the first four waves of the pandemic, simple/elective procedure volumes both fell and recovered at higher relative rates when compared with complex or cancer (non-elective) procedure volumes. Throughout the pandemic, there was a trend towards less cancelation of both elective and non-elective procedures with each successive wave. Overall, our findings characterize the COVID-19 pandemic as a cyclic disease that surgical specialties are learning to cope with over time.

## Appendices

**Table 2:** List of ICD-10-PCS procedure codes and CPT codes used in the study. ICD-10-CM diagnosis codes indicate the qualifying codes to present in addition to the procedure codes.

Procedures	Code Type	Code
Bariatric	CPT	43770, 43775
	ICD-10-PCS	0D164ZA, 0DB64Z3
	ICD-10-CM	E6601
Colectomy	ICD-10-PCS	0DBE4ZZ, 0DBF4ZZ, 0DBG4ZZ, 0DBH4ZZ, 0DBK4ZZ, 0DBL4ZZ, 0DBM4ZZ, 0DBN4ZX, 0DBN4ZZ, 0DBE0ZZ, 0DBF0ZZ, 0DBG0ZZ, 0DBH0ZZ, 0DBK0ZZ, 0DBL0ZZ, 0DBM0ZZ, 0DBN0ZX, 0DBN0ZZ, 0DTE4ZZ, 0DTF4ZZ, 0DTG4ZZ, 0DTH4ZZ, 0DTK4ZZ, 0DTL4ZZ, 0DTM4ZZ, 0DTN4ZZ, 0DTE0ZZ, 0DTF0ZZ, 0DTG0ZZ, 0DTH0ZZ, 0DTK0ZZ, 0DTL0ZZ, 0DTM0ZZ, 0DTN0ZZ
Rectal	ICD-10-PCS	0DBF0ZZ, 0DBH0ZZ, 0DBL0ZZ, 0DBM0ZZ, 0DBN0ZZ, 0DBP0ZZ, 0DTP4ZZ, 0DTP0ZZ
Lung lobectomy	ICD-10-PCS	0BTC4ZZ, 0BTD4ZZ, 0BTF4ZZ, 0BTG4ZZ, 0BTJ4ZZ, 0BTK4ZZ, 0BTL4ZZ, 0BTC0ZZ, 0BTD0ZZ, 0BTF0ZZ, 0BTG0ZZ, 0BTJ0ZZ, 0BTK0ZZ, 0BTL0ZZ, 0BBC4ZZ, 0BBD4ZZ, 0BBF4ZZ, 0BBG4ZZ, 0BBJ4ZZ, 0BBK4ZZ, 0BBL4ZZ, 0BBC0ZZ, 0BBD0ZZ, 0BBF0ZZ, 0BBG0ZZ, 0BBJ0ZZ, 0BBK0ZZ, 0BBL0ZZ, 0BBC4ZX, 0BBD4ZX, 0BBF4ZX, 0BBG4ZX, 0BBJ4ZX, 0BBK4ZX, 0BBL4ZX, 0BBC0ZX, 0BBD0ZX, 0BBF0ZX, 0BBG0ZX, 0BBJ0ZX, 0BBK0ZX, 0BBL0ZX
Hysterectomy	CPT	58550, 58552, 58553, 58554, 58541, 58542, 58543, 58544, 58570, 58571, 58572, 58573, 58260, 58262, 58263, 58270, 58290, 58291, 58292, 58294
	ICD-10-PCS	0UT90ZL, 0UT90ZZ, 0UTC0ZZ, 0UT9FZL, 0UT9FZZ, 0UT98ZL, 0UT98ZZ, 0UTC8ZZ, 0UT94ZL, 0UT94ZZ, 0UTC4ZZ, 0UT97ZL, 0UT97ZZ, 0UTC7ZZ
Incisional/ventral hernia	CPT	49652, 49653, 49654, 49655, 49656, 49657, 49560, 49561, 49565, 49566
	ICD-10-PCS	0WQF4ZZ, 0WUF4JZ, 0WUF4KZ, 0WUF47Z, 0WQF0ZZ, 0WUF0JZ, 0WUF0KZ, 0WUF07Z
Inguinal hernia	CPT	49650, 49651, 49492, 49495, 49496, 49500, 49501, 49505, 49507, 49520, 49521, 49525
	ICD-10-PCS	0YQ54ZZ, 0YQ64ZZ, 0YQA4ZZ, 0YU54JZ, 0YU54KZ, 0YU64JZ, 0YU64KZ, 0YUA4JZ, 0YQ50ZZ, 0YQ60ZZ, 0YQA0ZZ, 0YU50JZ, 0YU50KZ, 0YU60JZ, 0YU60KZ, 0YUA0JZ

**Table 3:** List of ICD-10-CM diagnosis code to differentiate cancer/complex and simple/elective cases.

Procedures	Diagnosis Type	ICD-10-CM Code	ICD-10-CM Code Description
All procedures	Cancer	C00 - C96	Malignant neoplasm
Incisional/ventral hernia	Complex	K430	Incisional hernia with obstruction, without gangrene
		K431	Incisional hernia with gangrene
		K433	Parastomal hernia with obstruction, without gangrene
		K434	Parastomal hernia with gangrene
		K436	Other and unspecified ventral hernia with obstruction, without gangrene
		K437	Other and unspecified ventral hernia with gangrene
	Simple	K432	Incisional hernia without obstruction or gangrene
		K435	Parastomal hernia without obstruction or gangrene
		K439	Ventral hernia without obstruction or gangrene
Inguinal hernia	Complex	K4000	Bilateral inguinal hernia, with obstruction, without gangrene, not specified as recurrent
		K4001	Bilateral inguinal hernia, with obstruction, without gangrene, recurrent
		K4010	Bilateral inguinal hernia, with gangrene, not specified as recurrent
		K4011	Bilateral inguinal hernia, with gangrene, recurrent
		K4030	Unilateral inguinal hernia, with obstruction, without gangrene, not specified as recurrent
		K4031	Unilateral inguinal hernia, with obstruction, without gangrene, recurrent
		K4040	Unilateral inguinal hernia, with gangrene, not specified as recurrent
		K4041	Unilateral inguinal hernia, with gangrene, recurrent
	Simple	K4020	Bilateral inguinal hernia, without obstruction or gangrene, not specified as recurrent
		K4021	Bilateral inguinal hernia, without obstruction or gangrene, recurrent
		K4090	Unilateral inguinal hernia, without obstruction or gangrene, not specified as recurrent
		K4091	Unilateral inguinal hernia, without obstruction or gangrene, recurrent
